# Readmission destination following cardiac surgery and its association with mortality outcomes: a population-based retrospective study

**DOI:** 10.1016/j.lanwpc.2024.101189

**Published:** 2024-09-04

**Authors:** Md Shajedur Rahman Shawon, Sanja Lujic, Yashutosh Joshi, Louisa Jorm

**Affiliations:** a Centre for Big Data Research in Health, University of New South Wales, Sydney, Australia; b Department of Cardiothoracic and Transplant Surgery, St Vincent's Hospital, Sydney, Australia

**Keywords:** Cardiac surgery, Readmission, Non-index readmission, CABG, Surgical aortic valve replacement

## Abstract

**Background:**

It is unclear how pre-surgery transfer relates to readmission destination among patients undergoing cardiac surgery and whether readmission to a hospital other than the operating hospital is associated with increased mortality.

**Methods:**

We analysed linked hospital and death records for residents of New South Wales, Australia, aged ≥18 years who had an emergency readmission within 30 days following coronary artery bypass graft (CABG) or surgical aortic valve replacement (SAVR) in 2003–2022. Mixed-effect multi-level modelling was used to evaluate associations of readmission destination with 30-day mortality, overall and stratified by pre-surgery transfer.

**Findings:**

Of 102,540 patients undergoing cardiac surgery (isolated CABG = 63,000, SAVR = 27,482, combined = 12,058), 28.7% (n = 29,398) had pre-surgery transfer, while the 30-day readmission rate was 14.7% (n = 14,708). During readmission, 35.7% (3499/9795) of those without pre-surgery transfer and 12.0% (590/4913) of those with pre-surgery transfer returned to the operating hospital. Among readmitted patients, 30-day mortality did not differ significantly for those who were readmitted to a non-index hospital, both overall (adjusted odds ratio [aOR] = 1.03 95% CI 0.75–1.41), and in analyses stratified by pre-surgery transfer (no transfer: aOR = 1.07, 95% CI 0.75–1.52; transfer: aOR = 0.88, 95% CI 0.45–1.72). Among patients who had pre-surgery transfer, 30-day mortality was similar among patients who were readmitted to the index operating hospital (reference), the initial admitting hospital (aOR = 1.00, 95% CI 0.50–2.00) or a third, different, hospital (aOR = 0.70, 95% CI 0.33–1.48).

**Interpretation:**

Although many Australian patients who are readmitted following cardiac surgery are readmitted to hospitals different to the operating or initial admitting hospital, such readmissions are not associated with increased mortality.

**Funding:**

This study was funded by a National Health and Medical Research Foundation of Australia (NHMRC) Project Grant (#1162833).

**Research in context**

**Evidence before this study**

We searched existing evidence on readmission destination following cardiac surgery, employing a variety of relevant search terms such as “readmission destination,” “non-index readmission,” “care fragmentation,” “coronary artery bypass graft (CABG),” “surgical aortic valve replacement,” “valve surgery,” and “cardiac surgery.” We found that two studies from the US indicated high proportion of patients were readmitted to hospitals different from where they underwent surgery (non-index hospitals), which was associated with an increased risk of subsequent mortality. This underscores a critical gap in post-discharge care continuity, largely due to the unfamiliarity of the treating clinicians with the patients and their surgical histories. Data on readmissions to non-operating hospitals for cardiac surgery patients remains scarce outside the US. This gap is particularly relevant in the Australian context, where the vast geography and a hybrid healthcare system, integrating both public and private sectors, could uniquely influence such readmission patterns.

**Added value of this study**

In this retrospective cohort study that included 102,540 patients undergoing cardiac surgery, only 27.8% of readmitted patients returned to the operating hospital. Significant predictor of readmission destination included having pre-surgery transfer, patient’s residence in regional/remote areas, lower socio-economic status, private insurance, and older age. Readmission to a hospital other than the operating hospital was not associated with increased mortality.

**Implications of all the available evidence**

In health systems serving geographically dispersed populations and featuring a mix of public and private healthcare services, it is inevitable that a large number of patients undergoing cardiac surgery at specialised facilities in major cities will be readmitted to a different hospital than the one where their surgery took place. Therefore, it is essential to consider factors such as the geographical distribution of the population, the interplay between public and private healthcare provisions, and the care pathways that include pre-surgery transfers to specialised centres. These elements will play a crucial role in understanding the implications of non-index hospital readmissions following cardiac surgery.

## Introduction

More than one in ten patients undergoing coronary artery bypass graft (CABG) and surgical valve replacement procedures experience unplanned readmissions,^1^ which are associated with poorer outcomes, including increased risk of mortality and substantial financial burden on health systems.^2^ Despite the potentially critical role of readmissions in managing complications resulting following cardiac surgery, factors that drive poor outcomes in readmitted patients remain insufficiently studied. Two US studies^3^^,^^4^ found that a substantial number of readmissions after cardiac surgery occurred at a hospital different to the operating hospital (i.e., a non-index hospital), leading to a notable rise in mortality risk. This suggests lack of continuity of care during the post-discharge period, as treating clinicians may lack familiarity with patients and their procedures.^3^^,^^5^ Nonetheless, there is limited data outside the US on non-operating hospital readmissions for cardiac surgery patients.

Australia's healthcare system services a geographically dispersed population, with both public and private hospitals providing specialized services, which can lead to high rates of readmission to non-index hospitals.^5^^,^^6^ Our study aims to investigate the occurrence of non-index readmissions in Australian cardiac surgery patients and its impact on mortality. Moreover, given that many patients are transferred from a referral hospital to the operating hospital,^7^ we explore readmission destinations and outcomes separately for patients according to pre-surgery transfer and readmission causes.

## Methods

### Data source and study population

In this study, we used individual level linked hospital data from New South Wales (NSW) Admitted Patient Data Collection (APDC) and mortality data from the NSW Register of Births, Deaths, and Marriages (RBDM). In the APDC data, primary and secondary diagnoses are coded using the International Classification of Diseases and Related Problems 10th Revision, Australian Modification (ICD-10-AM),^8^ whereas any procedures performed are coded according to the Australian Classification of Health Interventions (ACHI).^9^

We included patients aged 18 years or over with a procedure code for CABG or surgical valve replacement (relevant ACHI codes are given in Supplementary Table S1) between 2003 and 2022. We excluded admissions with potential linkage errors (<0.02% of total), such as discharges before admissions or admissions after death dates. If any patient had two cardiac surgeries within 30 days, the latter was considered as readmission. APDC data includes episodes of care which can end in transfer, discharge or death, and therefore we considered multiple, contiguous episodes as a single, acute period of hospital stay. We also considered hospital episodes commencing on the same day of separation from another hospital episode as the same acute period of hospital stay, regardless of the mode of separation at the previous episode. We considered a pre-surgery hospital transfer when during a single period of hospital stay a patient was first admitted to one hospital and then transferred to another where cardiac surgery was performed. For all the patients included in our study, we identified the operating hospital. Additionally, for those who experienced a pre-surgery hospital transfer, we identified the hospital where the patient was initially admitted during that specific hospital stay (i.e., admitting hospital). The NSW Population and Health Services Research Ethics Committee granted ethical approval for this study (ref: 2019/ETH00436). No patient consent was required as we used routinely collected administrative data, but appropriate approvals were obtained from the relevant data custodians for ethics approval.

### 30-day readmission

We identified all emergency hospital readmissions within 30 days of the discharge following the cardiac surgery. If there were multiple emergency readmissions within 30 days, only the first readmission was considered for analysis. The hospital where the patient was first admitted during the readmission was considered as the readmission destination. We categorized readmission destinations into two groups: readmission to the operating hospital and readmission to a different hospital. Additionally, for those with pre-surgery hospital transfer, we considered three categories for readmission destination: readmission to the operating hospital, readmission to the initial admitting hospital, and readmission to a different hospital.

Based on prior works conducted by the Australian Institute for Health and Welfare (AIHW)^10^ and the NSW Bureau of Health Information (BHI),^11^ we categorized readmissions into three groups based on the principal diagnosis: cardiovascular conditions; conditions potentially related to hospital care; and other conditions.

### Outcomes

The primary outcome of our study was 30-day all-cause mortality which was defined as any death that occurred within 30 days from the readmission date. The secondary outcome was 1-year all-cause mortality, defined as any death within one year from the readmission date. We obtained information on death from the linked NSW RBDM data.

### Covariates

From the initial hospitalisation involving cardiac surgery, we collected data regarding the patient's age at the time of surgery, sex, type of surgery (i.e., isolated CABG, isolated valve, or combined), patient's residential remoteness, socioeconomic status, emergency admission, pre-surgery hospital transfer, private health insurance, length of stay, and whether the hospital was publicly or privately funded. We identified any comorbidities by examining the primary and secondary diagnosis codes recorded during the initial hospitalisation and any hospital records from up to two years prior (see Supplementary Table S1). Patient's residential remoteness was categorized using the Accessibility/Remoteness Index of Australia score for the patient's Statistical Area Level 2 (SA2) of residence,^12^ which was grouped into two categories: major cities and regional/remote areas. The Socio-Economic Index for Areas, assigned to the patient's SA2 of residence, was used to assign socioeconomic status, and this was split into five population quintiles.^13^

### Statistical analysis

We characterised patients who underwent cardiac surgery, stratified by whether they had a pre-surgery hospital transfer or not. To test differences in characteristics, we used the 2-sample Student's t-test or Mann–Whitney test for continuous variables and chi-square test for categorical variables, as appropriate. Among the readmitted patients, we estimated the proportion of patients readmitted to a hospital different to the operating hospital. We then compared patient and hospital characteristics according to readmission destination (same operating hospital vs. different hospital). Among these patients, we also identified the factors associated with readmission to a hospital different to the index operating hospital by building separate multiple logistic regression models for each factor with adjustment for patient case-mix (i.e., age, sex, and comorbidities).

To assess the independent association between readmission destination and subsequent mortality, we constructed multi-level mixed-effect logistic regression models with adjustments for age at index surgery, sex, socioeconomic status, remoteness of residence, private insurance, emergency admission, procedure type, readmission reason, operating hospital type and comorbidities. We included operating hospitals as a random effect intercept to account for variation across hospitals. We estimated adjusted odds ratio (aOR) with 95% confidence intervals (CIs) for associations of non-index readmission with 30-day mortality and 1-year mortality. We repeated these analyses stratified according to reason for readmission category and pre-surgery hospital transfer status. For those who had pre-surgery transfer, we additionally explored whether mortality was similar among patients who were readmitted to the index operating hospital (reference), the initial admitting hospital or a third, different, hospital. Missing values were treated as a separate category. To address concerns of potential overfitting, covariates were added sequentially during the statistical analysis, and the OR estimates were found to remain stable, indicating that overfitting did not substantially impact the model results.

All statistical tests were two-sided and were conducted using Stata version 16.0 (College Station, Texas). P-values < 0.05 were considered statistically significant.

### Role of the funding sources

The funding authorities had no role in the study design, data collection, data analysis, interpretation, or writing of the manuscript.

## Results

**Fig. 1:**
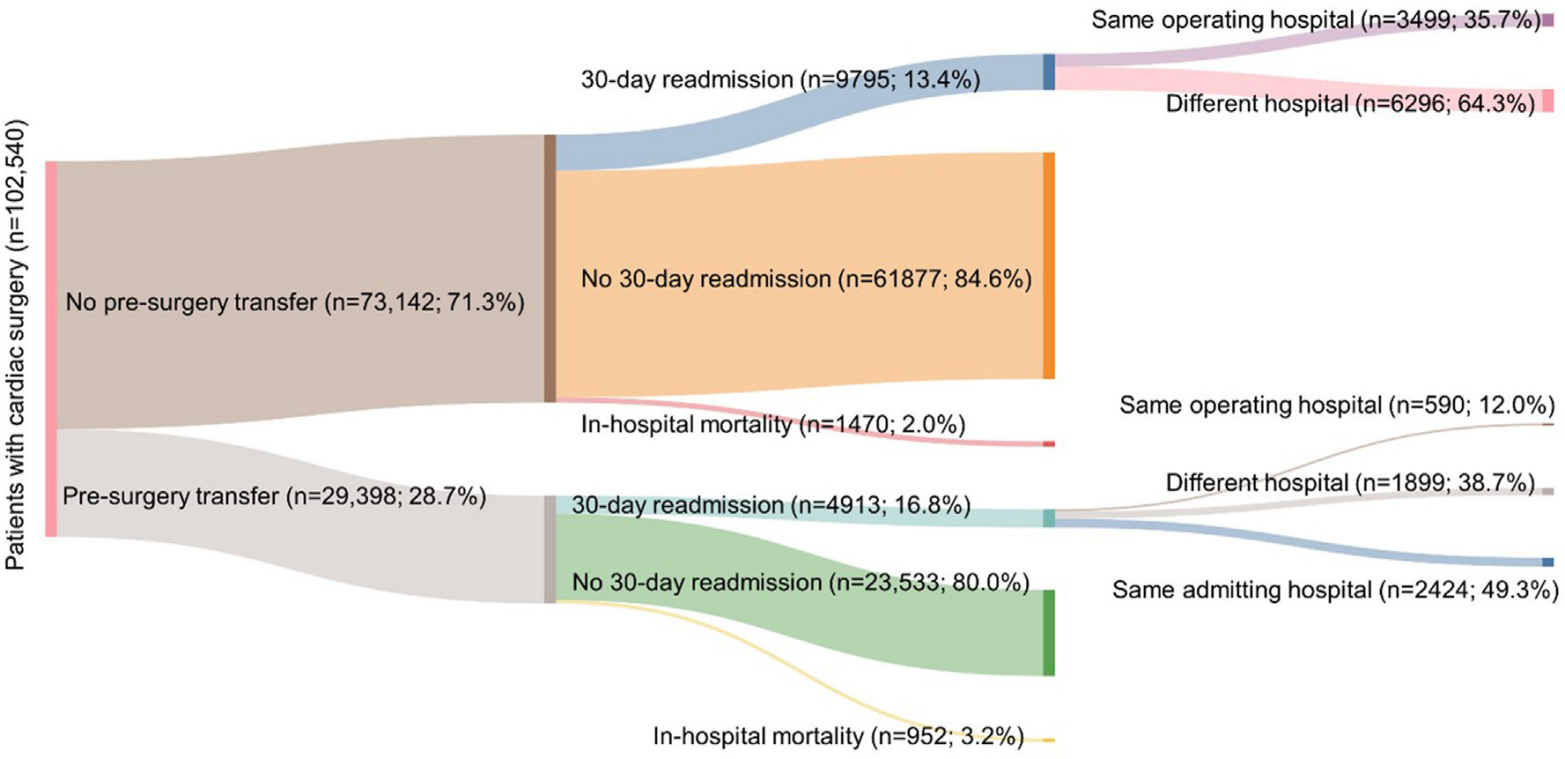
Flowchart showing outcomes for patients undergoing cardiac surgery, according to pre-surgery transfer status.

During 2003–2012, there were 102,540 cardiac surgeries (isolated CABG = 63,000, isolated valve = 27,482 and combined = 12,058) across 49 public and private hospitals. The mean (standard deviation, SD) age of patients undergoing cardiac surgery was 67.6 (11.7) years, and a quarter (26.0%, n = 26,683) of all patients were female. Two-thirds of patients (n = 76,567) were from major cities, and 42.9% (n = 43,988) had private health insurance. Nearly three quarters (71.3%, n = 73,142) of all patients were directly admitted to the operating hospital whereas 28.7% had pre-surgery hospital transfer. Table 1 shows the patient characteristics according to pre-surgery transfer status. Compared to patients without pre-surgery transfer, those with pre-surgery transfer were more likely to undergo isolated CABG (75.4% [n = 22,166/29,398] vs. 55.8% [n = 40,813]), reside in regional/remote areas (38.1% [n = 11,186/29,398] vs. 20.2% [n = 14,787/73,142]), have emergency admission (77.2% [n = 22,693/29,398] vs. 17.7% [n = 12,910/73,142]), have more comorbidities, and undergo the surgery in a public hospital (65.3% [n = 19,184/29,398] vs. 54.9% [n = 40,159/73,142]). Patients with pre-surgery transfer were also more likely to die in-hospital (3.2% [n = 952/29,398] vs. 2.0% [n = 1470/73,142]) and be readmitted within 30 days of discharge (17.3% [n = 4913/29,398] vs. 13.7% [n = 9795/73,142]) compared to those without pre-surgery transfer (Fig. 1).

**Table 1:** Selected characteristics of patients undergoing cardiac surgery (2003–2022), overall and by pre-surgery transfer status.

	No. (%) of patients			
	All patients	Did not have pre-surgery transfer	Had pre-surgery transfer	P-value^a^
No. of procedures	102,540 (100.0)	73,142 (71.3)	29,398 (28.7)	
Procedure type				<0.0001
CABG	75,058 (73.2)	49,907 (68.2)	25,151 (85.6)	
Valve	27,482 (26.8)	23,235 (31.8)	4247 (14.4)	
Patient age, years mean (SD)	67.6 (11.7)	67.6 (11.8)	67.7 (11.5)	0.64
Female	26,683 (26.0)	19,489 (26.6)	7194 (24.5)	<0.0001
Socio-economic status				<0.0001
Q1–most disadvantaged	20,313 (19.8)	13,206 (18.1)	7107 (24.2)	
Q2	22,207 (21.7)	13,984 (19.1)	8223 (28.0)	
Q3	18,623 (18.2)	13,788 (18.9)	4835 (16.4)	
Q4	13,629 (13.3)	10,034 (13.7)	3595 (12.2)	
Q5–least disadvantaged	19,039 (18.6)	15,558 (21.3)	3481 (11.8)	
Missing	8729 (8.5)	6572 (9.0)	2157 (7.3)	
Remoteness of residence				<0.0001
Major cities	76,567 (74.7)	58,355 (79.8)	18,212 (61.9)	
Regional/remote areas	25,973 (25.3)	14,787 (20.2)	11,186 (38.1)	
Private insurance	43,998 (42.9)	33,478 (45.8)	10,520 (35.8)	<0.0001
Emergency admission	35,603 (34.7)	12,910 (17.7)	22,693 (77.2)	<0.0001
Length of stay (days), median (IQR)	13 (8,21)	10 (8,17)	19 (14,28)	<0.0001
Comorbidity profile				
Diabetes	31,079 (30.3)	21,136 (28.9)	9943 (33.8)	<0.0001
Hypertension	75,292 (73.4)	52,182 (71.3)	23,110 (78.6)	<0.0001
Myocardial infarction	28,902 (28.2)	12,883 (17.6)	16,019 (54.5)	<0.0001
Cardiac arrhythmias	57,292 (55.9)	40,695 (55.6)	16,597 (56.5)	<0.0001
Valvular disease	43,675 (42.6)	34,790 (47.6)	8885 (30.2)	<0.0001
Congestive heart failure	20,983 (20.5)	13,480 (18.4)	7503 (25.5)	0.024
Cardiogenic shock	2296 (2.2)	1316 (1.8)	980 (3.3)	0.082
Peripheral vascular disease	10,462 (10.2)	7404 (10.1)	3058 (10.4)	<0.0001
Stroke	8327 (8.1)	5365 (7.3)	2962 (10.1)	<0.0001
Chronic pulmonary disease	11,986 (11.7)	8080 (11.0)	3906 (13.3)	<0.0001
Pulmonary circulation disorders	6378 (6.2)	4452 (6.1)	1926 (6.6)	<0.0001
Chronic kidney disease	13,941 (13.6)	9180 (12.6)	4761 (16.2)	0.012
Liver disease	2897 (2.8)	1881 (2.6)	1016 (3.5)	0.062
Rheumatoid arthritis and collagen vascular disease	9393 (9.2)	6596 (9.0)	2797 (9.5)	<0.0001
Cancer	3223 (3.1)	2280 (3.1)	943 (3.2)	<0.0001
Index hospital funding type				<0.0001
Public	59,343 (57.9)	40,159 (54.9)	19,184 (65.3)	
Private	43,197 (42.1)	32,983 (45.1)	10,214 (34.7)	
In-hospital mortality	2422 (2.4)	1470 (2.0)	952 (3.2)	<0.0001
30-day readmission	14,708 (14.7)	9795 (13.7)	4913 (17.3)	<0.0001

Values are in n (%) except otherwise mentioned.

a Chi-square test was conducted for categorical variables, whereas T-test or Mann–Whitney test was used for continuous variables.

The 30-day readmission rate was 14.7% (14,708/102,540). Among those readmitted within 30 days, 27.8% (4089/14,708) returned to the index operating hospital. The rate of index readmission was much higher among patients without pre-surgery transfer (35.7%, n = 3499/14,708) than those with pre-surgery transfer (12.0%, n = 590/4913) (Fig. 1). Among patients with pre-surgery transfer, almost half (49.3%, n = 2424/4913) were readmitted to the initial admitting hospital, while 38.7% (n = 1899/4913) were readmitted to a third, different, hospital (Fig. 1). Patients readmitted to a non-index hospital tended to be older, female, residents of regional/remote areas, privately insured, and more likely to have the surgery at a private hospital (Table 2). They were also more likely to have medical comorbidities such as cardiac arrhythmias, valvular disease, congestive heart failure, peripheral vascular disease, and pulmonary circulation disorders (Table 2).

**Table 2:** Selected characteristics of patients readmitted within 30 days following cardiac surgery (2003–2022), overall and by pre-surgery transfer status.

	No. (%) of readmission				
	Did not have pre-surgery transfer		Had pre-surgery transfer		
	Same operating hospitals	Different hospital	Same operating hospital	Same admitting hospital	Different hospital
No. of procedures	3499 (35.7)	6296 (64.3)	590 (12.0)	2424 (49.3)	1899 (38.7)
Procedure type					
CABG	2433 (69.5)	3990 (63.4)	498 (84.4)	2054 (84.7)	1523 (80.2)
Valve	1066 (30.5)	2306 (36.6)	92 (15.6)	370 (15.3)	376 (19.8)
Patient age, years mean (SD)	67.1 (12.3)	69.3 (12.1)	65.8 (12.8)	68.4 (11.5)	69.6 (11.8)
Female	976 (27.9)	1980 (31.4)	163 (27.6)	680 (28.1)	539 (28.4)
Socio-economic status					
Q1–most disadvantaged	570 (16.3)	1348 (21.4)	143 (24.2)	618 (25.5)	573 (30.2)
Q2	452 (12.9)	1544 (24.5)	111 (18.8)	773 (31.9)	526 (27.7)
Q3	858 (24.5)	1058 (16.8)	123 (20.8)	396 (16.3)	299 (15.7)
Q4	438 (12.5)	838 (13.3)	69 (11.7)	274 (11.3)	191 (10.1)
Q5–least disadvantaged	765 (21.9)	1048 (16.6)	78 (13.2)	203 (8.4)	187 (9.8)
Missing	416 (11.9)	460 (7.3)	66 (11.2)	160 (6.6)	123 (6.5)
Remoteness of residence					
Major cities	3320 (94.9)	4315 (68.5)	519 (88.0)	1436 (59.2)	985 (51.9)
Regional/remote areas	179 (5.1)	1981 (31.5)	71 (12.0)	988 (40.8)	914 (48.1)
Private insurance	891 (25.5)	2696 (42.8)	119 (20.2)	600 (24.8)	611 (32.2)
Emergency admission	1429 (40.8)	711 (11.3)	473 (80.2)	2254 (93.0)	1264 (66.6)
Length of stay (days), median (IQR)	12 (8,21)	11 (8,18)	20 (14,29)	21 (16,31)	21 (15,30)
Comorbidity profile					
Diabetes	1258 (36.0)	1892 (30.1)	249 (42.2)	938 (38.7)	709 (37.3)
Hypertension	2546 (72.8)	4588 (72.9)	481 (81.5)	1953 (80.6)	1516 (79.8)
Myocardial infarction	950 (27.2)	1021 (16.2)	322 (54.6)	1486 (61.3)	945 (49.8)
Cardiac arrhythmias	2048 (58.5)	4050 (64.3)	338 (57.3)	1470 (60.6)	1231 (64.8)
Valvular disease	1580 (45.2)	3503 (55.6)	180 (30.5)	788 (32.5)	711 (37.4)
Congestive heart failure	813 (23.2)	1436 (22.8)	165 (28.0)	748 (30.9)	614 (32.3)
Cardiogenic shock	69 (2.0)	89 (1.4)	23 (3.9)	76 (3.1)	58 (3.1)
Peripheral vascular disease	363 (10.4)	722 (11.5)	62 (10.5)	269 (11.1)	244 (12.8)
Stroke	293 (8.4)	562 (8.9)	61 (10.3)	254 (10.5)	225 (11.8)
Chronic pulmonary disease	499 (14.3)	919 (14.6)	88 (14.9)	415 (17.1)	316 (16.6)
Pulmonary circulation disorders	231 (6.6)	467 (7.4)	35 (5.9)	188 (7.8)	156 (8.2)
Chronic kidney disease	571 (16.3)	988 (15.7)	109 (18.5)	475 (19.6)	389 (20.5)
Liver disease	133 (3.8)	193 (3.1)	23 (3.9)	80 (3.3)	74 (3.9)
Rheumatoid arthritis and collagen vascular disease	325 (9.3)	684 (10.9)	61 (10.3)	235 (9.7)	238 (12.5)
Cancer	117 (3.3)	215 (3.4)	12 (2.0)	79 (3.3)	71 (3.7)
Index hospital funding type					
Public	2931 (83.8)	3514 (55.8)	518 (87.8)	1856 (76.6)	1286 (67.7)
Private	568 (16.2)	2782 (44.2)	72 (12.2)	568 (23.4)	613 (32.3)
Readmission parameters					
Readmission reason					
Cardiovascular conditions	974 (27.8)	2013 (32.0)	142 (24.1)	832 (34.3)	447 (23.5)
Potentially related to hospital care	1606 (45.9)	2288 (36.3)	277 (46.9)	952 (39.3)	620 (32.6)
Other conditions	919 (26.3)	1995 (31.7)	171 (29.0)	640 (26.4)	832 (43.8)
Length of stay (days), median (IQR)	4 (2,8)	4 (1,8)	5 (2,10)	4 (1,8)	5 (2,12)
30-day mortality	59 (1.7)	123 (2.0)	11 (1.9)	53 (2.2)	28 (1.5)
1-year mortality	200 (5.7)	427 (6.8)	38 (6.4)	208 (8.6)	146 (7.7)

Values are in n (%) except otherwise mentioned.

Characteristics were present at the index hospitalisation with cardiac surgery.

Less than one third of all readmissions (29.9%, n = 4408/14,708) were due to cardiovascular conditions, whereas 39.1% (n = 5743/14,708) were due to conditions potentially related to hospital care and 31.0% (n = 4557/14,708) were due to other conditions (Table 2). Supplementary Table S2 lists the top 10 ICD-10 codes for these three categories of readmission. Patients readmitted due to conditions potentially related to hospital care, which included infections, pneumonia and embolisms, had a higher likelihood of being admitted to the index operating hospital.

**Fig. 2:**
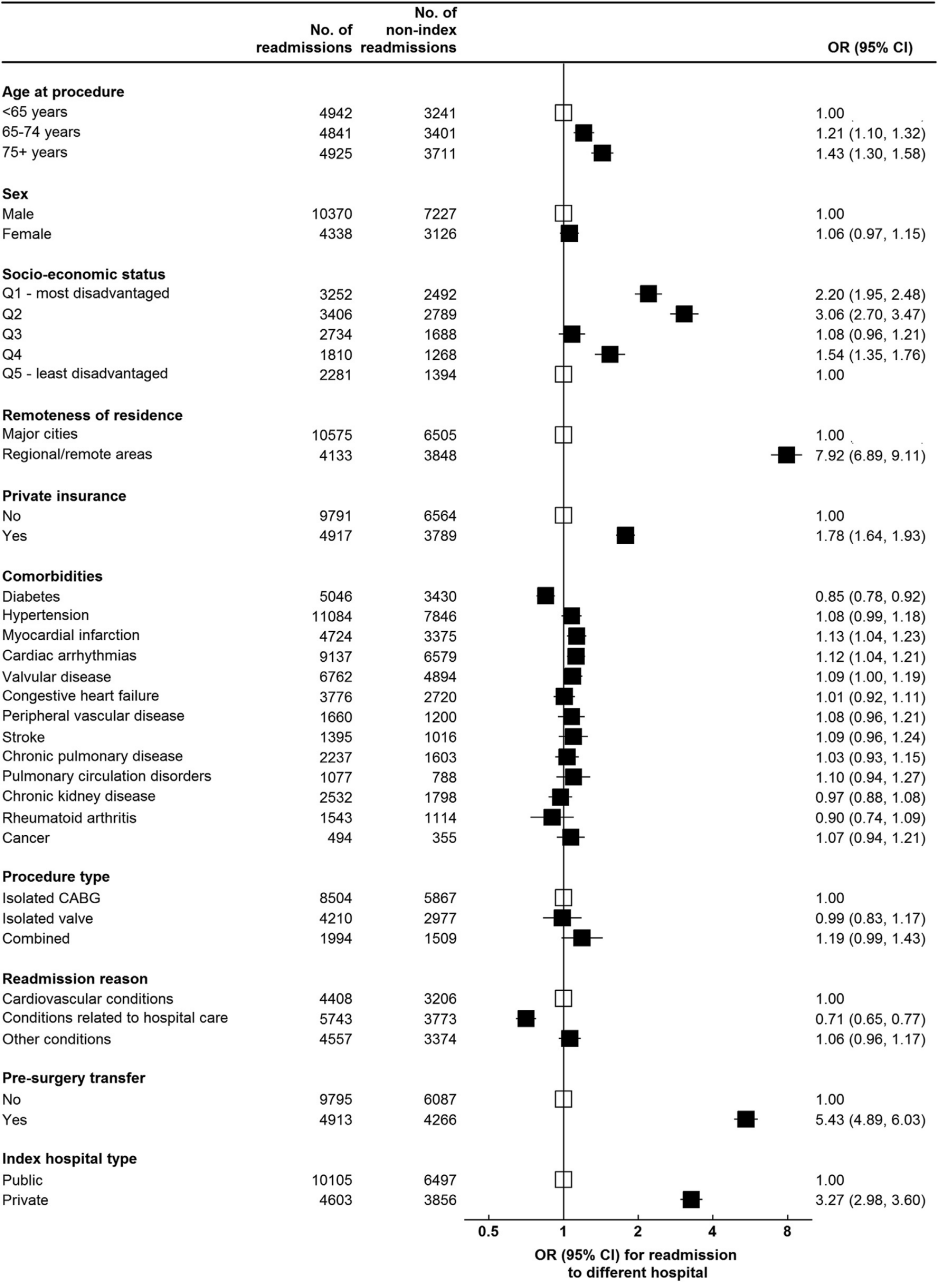
Factors associated with readmission to a non-operating hospital following cardiac surgery. Odds ratios (ORs) with 95% confidence intervals (CIs) are estimated from separate multiple logistic regression models for each factor presented in the figure, with adjustments for age, sex, and comorbidities. ORs for each comorbidity were adjusted for all other comorbidities.

Readmission to a non-index hospital was strongly associated with remoteness of patient’s residence (aOR for regional/rural areas vs. major cities = 7.92, 95% CI 6.89–9.11), having pre-surgery hospital transfer (aOR = 5.43, 95% CI 4.89–6.03), and private index hospital (aOR = 3.27, 95% CI 2.98–3.60) (Fig. 2). Other factors associated with non-index readmission included older age (aOR 75+ years vs. <65 years = 1.43, 95% CI 1.30–1.58), higher socioeconomic status (aOR for most disadvantaged vs. least disadvantaged = 2.20, 95% CI 1.95–2.48), and having private insurance (aOR = 1.78, 95% CI 1.64–1.93). Readmission due to conditions potentially related to hospital care was negatively associated with readmission to a non-index hospital (aOR = 0.71, 95% CI 0.65–0.77).

**Fig. 3:**
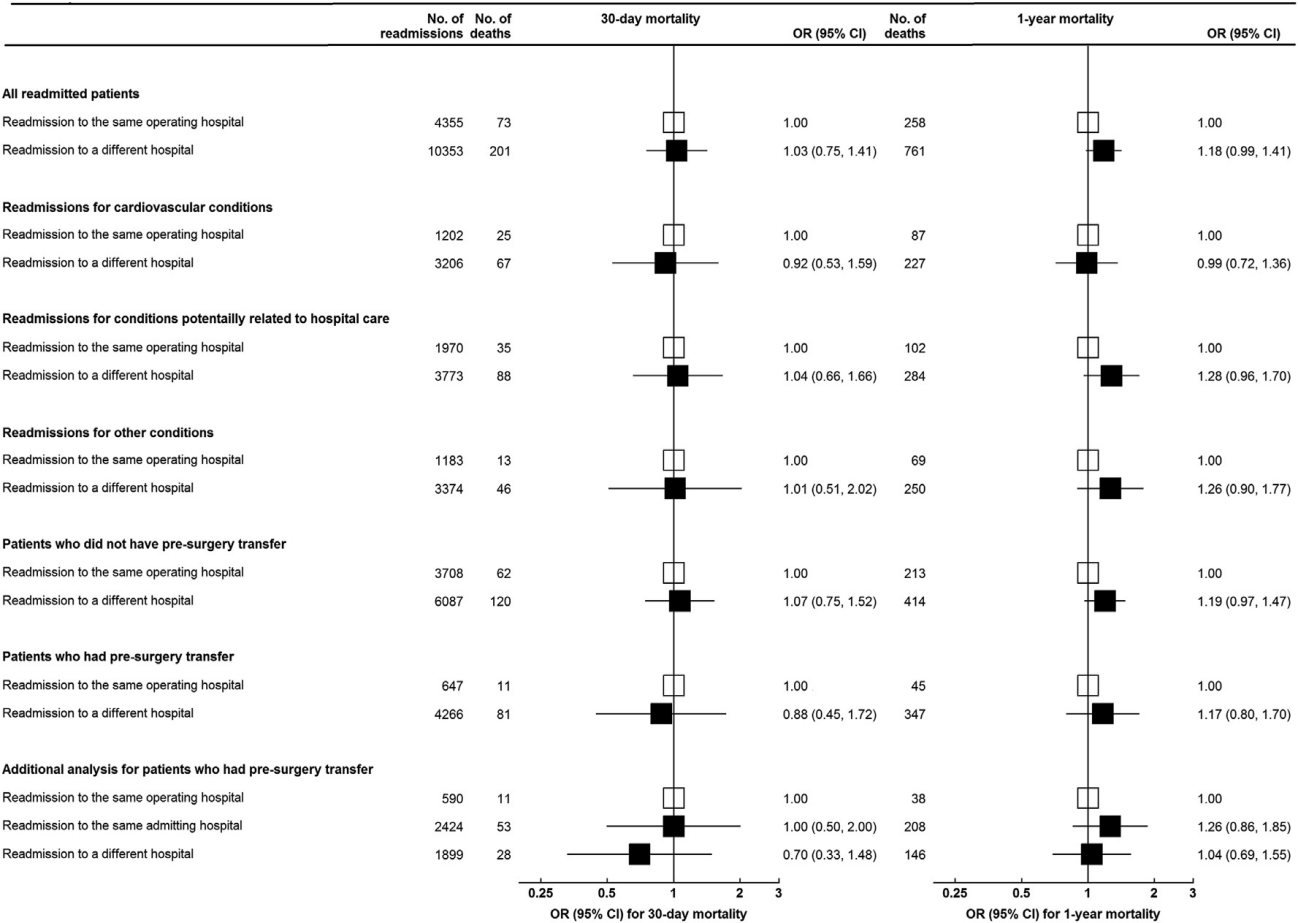
Associations of readmission destination with 30-day and 1-year mortality, according to readmission causes and pre-surgery hospital transfer status. Odds ratios (ORs) with 95% confidence intervals (CIs) are estimated from mixed-effect multi-level logistic regression models with a random intercept for operating hospital and with adjustments for age at index surgery, sex, socioeconomic status, remoteness of residence, private insurance, emergency admission, procedure type, readmission reason, operating hospital type and comorbidities.

Rates of 30-day and 1-year mortality among readmitted patients were 1.9% (n = 274/14,708) and 6.9% (n = 1019/14,708), respectively (Table 2). The rate of 30-day mortality was comparable among patients readmitted to the index operating hospital and those readmitted a non-index hospital (aOR = 1.03, 95% CI 0.75–1.41) (Fig. 3). Patients readmitted to a non-index hospital had higher odds of 1-year mortality, but the association did not reach statistical significance (aOR = 1.18, 95% CI 0.99–1.41). We did not observe any significant association between readmission to a non-index hospital and 30-day mortality when we looked separately among patients with and without pre-surgery hospital transfer (aOR = 1.07 [95% CI 0.75–1.52] and aOR = 0.88 [95% CI 0.45–1.72], respectively). Additionally, among patients who had pre-surgery transfer, 30-day mortality was similar among patients who were readmitted to the operating hospital (reference), the initial admitting hospital (aOR = 1.00 [95% CI 0.50–2.00]) or a third, different, hospital (aOR = 0.70 [95% CI 0.33–1.48]) (Fig. 3). The associations were also similar by readmission cause (cardiovascular conditions: aOR = 0.92 [0.53–1.59]; conditions related to hospital care (aOR = 1.04 [0.66–1.66]; other conditions: aOR = 1.01 [0.51–2.02]). Stratified analysis by readmission cause and pre-surgery hospital transfer status were similar for 1-year mortality (Fig. 3).

## Discussion

In this population-wide study of patients undergoing cardiac surgery, we found that nearly 1 in every 7 patients was readmitted within 30 days of hospital discharge and 27.8% of them returned to the index hospital where the surgery took place. Readmission to a non-index hospital was particularly driven by rural and remote patient residence, pre-surgery transfer, private insurance and privately funded index hospital. Among readmitted patients, 30-day and 1-year mortality were similar for those readmitted to index and non-index hospitals. Additionally, when looked separately by readmission cause and pre-surgery hospital transfer status, no significant association was observed between non-index readmission and mortality outcomes.

The 30-day readmission rate following cardiac surgery (14.7%) in our study is consistent with rates reported in previous studies.^1^^,^^3^^,^^4^^,^^14^ A recent meta-analysis reported a pooled 30-day readmission rate following CABG surgery of 12.9% (95% CI 11.3%–14.4%).^1^ Our study also found that 28.7% of all patients who underwent cardiac surgery had pre-surgery inter-hospital transfer, which was much lower than the rate of pre-surgery transfer (57.9%) reported in a recent US study.^7^ However, the US study restricted their analysis to only non-elective cardiac surgeries, while we included both elective and non-elective surgeries.

We found that nearly three-quarters (72.2%) of all readmitted patients went to a non-index hospital. While no previous studies from Australia have specifically explored the rate of non-index readmission following cardiac surgery, a few US studies provide some context.^3^^,^^4^ For example, Hirji et al.^4^ found that 23% and 26% of all patients readmitted within 30 days following CABG and surgical aortic valve replacement, respectively, were readmitted to non-index hospitals. Another study of US Medicare beneficiaries^3^ reported that 34.2% of all CABG patients readmitted within 30 days were readmitted to non-index hospitals. The much higher rates of non-index readmission in our study likely reflect Australia’s dispersed geography, as well as the role of private hospitals as major providers of elective surgery in the Australian health system.^15^^,^^16^

Readmission destination following cardiac surgery was greatly influenced by patients’ area of residence, with only 6.1% of patients from regional/rural areas were readmitted to the index hospital, compared to 36.3% for patients from major cities. Furthermore, given the higher rate of pre-surgery inter-hospital transfer among patients from regional/remote areas (43.1% vs. 23.8% for patients from major cities), it appears that they tend to receive surgical intervention at higher-level facilities during their index hospitalisation, but choose facilities closer to home for quicker access to care during readmission. This interpretation is also supported by the fact that nearly half of the patients who experienced pre-surgery hospital transfer were readmitted to the initial admitting hospital instead of the index operating hospital, indicating the influence of proximity on hospital choice.

We identified several additional factors associated with readmission to a non-index hospital, including older patient, higher socioeconomic status, and private insurance. The US study of Medicare beneficiaries^4^ also reported similar independent predictors of non-index readmission. The association between private insurance and higher socioeconomic status and non-index readmission is likely to reflect greater access to private hospitals for surgery. Unlike US private hospitals, Australian private hospitals primarily focus on elective procedures and typically lack emergency departments.^16^ Consequently, Australians who undergo cardiac surgery at a private hospital are unlikely to be readmitted to the same private hospital in case of an emergency. We also observed that compared to patients readmitted for cardiovascular conditions or other conditions, those readmitted for conditions potentially related to initial hospital care were more likely to return to the index operating hospital. This finding suggests that continuity of care, with its inherent advantages such as familiarity with initial surgical details, is a factor that drives readmission destination for these patients. However, we found no association between non-index readmission and mortality outcomes in stratified analysis according to cause of readmission.

Our study indicated that 30-day mortality rates were comparable between patients readmitted to the operating hospital and those readmitted to a different hospital. This similarity remained when we separately examined patients with and without pre-surgery hospital transfers. Despite the high rate of non-index readmission in our study, it was reassuring to observe that mortality rates were similar regardless of the destination of readmission. Our findings contrast with those of the two previous US studies that reported a higher likelihood of mortality associated with non-index readmission following cardiac surgery.^3^^,^^4^ This discrepancy may suggest better coordination of care across hospitals in Australia, perhaps facilitated by the fact that many Australian surgeons have appointments at both public and private hospitals. However, the US studies used older data (2015 and prior) and reported higher in-hospital mortality rates than our study, so may not reflect contemporary practice in the US.

Our study has some limitations. The administrative data that we used lack detailed information about specific surgical techniques and other clinical variables. We could not identify individual surgeons, so could not explore the potential impacts of their affiliations across multiple hospitals. Although our study leveraged best-practice multi-level mixed-effect modelling with a random intercept for the operating hospital and adjustments for a wide range of patient-level and hospital-level covariates, residual confounding may still exist due to unmeasured variables associated with mortality. However, we used a large, statewide dataset from the most populous state in Australia, along with robust statistical methodologies, to minimize bias and enhance the generalizability of our findings.

In conclusion, in our cohort of patients who were readmitted within 30 days following cardiac surgery, nearly three-quarters were readmitted to a non-index hospital. Factors such as the patient's residential remoteness, pre-surgery transfers, type of hospital performing the surgery and the reason for readmission significantly influenced the destination of readmission. Patients readmitted to index and non-index hospitals had similar 30-day and 1-year mortality.

## Contributors

MS and LJ conceptualised and designed the study. MS led the data curation, formal analysis, interpretation of findings, and visualisation. SL verified the data. MS and LJ wrote the original draft of the manuscript, with SL and YJ contributing to its revision. All authors contributed to the interpretation of findings. MS, SL, and LJ confirm they had full access to all the data in the study. All authors accept responsibility for submitting this manuscript for publication.

## Data sharing statement

The data underlying this article cannot be shared publicly because the ethics approval for the study does not allow us to share the data publicly. The aggregated data will be shared on reasonable request to the corresponding author.

## Declaration of interests

LJ received funding support from the Australian National Health and Medical Research Council Project Grant for this work, while the other authors declare no competing financial interests or personal relationships that could have influenced the work reported in this paper.
