# Supplementary material for: Readmission destination following cardiac surgery and its association with mortality outcomes: a population-based retrospective study
Source: Lancet Reg Health West Pac. 2024 Sep 4;51:101189. doi: 10.1016/j.lanwpc.2024.101189 (PMC11408007; doi:10.1016/j.lanwpc.2024.101189)
Supplement: Supplementary Tables S1 and S2 [file mmc1.pdf]

Readmission destination following cardiac surgery and its association with mortality outcomes

Supplementary materials

Table of contents

Supplementary Table S1. Detailed list of codes

Supplementary Table S2: Top 10 reasons for readmission according to readmission category

Supplementary Table S1. Detailed list of codes

Variable	Description
Procedures	***Australian Classification of Health Interventions (ACHI) codes***[1]
Surgical valve replacement	38456-10, 38483-00, 38270-01, 38480-00, 38481-00, 38488-00, 38488-01, 38489-00, 38489-01, 38456-15, 38653-04, 38475-02, 38477-02, 38487-00, 38485-01, 38270-02, 38480-01, 38481-01, 38475-00, 38477-00, 38488-02, 38488-03, 38489-02, 38485-00, 38456-16, 38653-05, 38456-11, 38480-02, 38481-02, 38475-01, 38477-01, 38488-04, 38488-05, 38489-03, 38456-17, 38653-06, 38456-01, 38270-03, 38488-06, 38488-07, 38489-04, 38489-05, 38456-18, 38653-07
Coronary artery bypass grafting (CABG)	38497-00, 38497-01, 38497-02, 38497-03, 38497-04, 38497-05, 38497-06, 38497-07, 38500-00, 38500-01, 38500-02, 38500-03, 38500-04, 38500-05, 38503-00, 38503-01, 38503-02, 38503-03, 38503-04, 38503-05, 90201-00, 90201-01, 90201-02, 90201-03
Medical conditions	***International Statistical Classification of Diseases and Related Health Problems, 10th Revision, Australian Modification (ICD-10-AM) codes and definitions***[1,2]
Myocardial infarction (MI)	I21
Diabetes	E10, E11, E12, E13, E14
Hypertension	I10, U82.3, I11, I12, I13, I15
COPD	J43, J44, U83.2
Congestive heart failure	I43, I50, I09.9, I11.0, I13.0, I13.2, I42.0, I42.5, I42.6, I42.7, I42.8, I42.9, P29.0, U82.2
Chronic kidney disease	N18, U87.1
Cardiomyopathy	I42
Coronary artery disease	I20, I21, I22, I24, I25, U82.1
Sleep apnoea	G47.3
Obesity	E66, U78.1
Stroke	G45, G46, H34.0, I60, I61, I62, I63, I64, I65, I66, I67, I68, I69
Peripheral vascular disease	I70, I71, I73.1, I73.8, I73.9, I77.1, I79.0, I79.2, K55.1, K55.8, K55.9, Z95.8, Z95.9, U82.2
Atrial fibrillation	I48

Sources:
[1] Australian Institute of Health and Welfare (2021). Better Cardiac Care measures for Aboriginal and Torres Strait Islander people: sixth national report 2021. Canberra: AIHW.
[2] Australian Coding Standard (ACS) 0003 Supplementary codes for chronic conditions (https://www.ihacpa.gov.au/) were included to account for coding standards changes in 2015 in Australia

Supplementary Table S2: Top 10 reasons for readmission according to readmission category

Readmission reason category	Frequency
**Cardiovascular conditions**	
Congestive heart failure I50.0	769
Chest pain, unspecified R07.4	691
Atrial fibrillation and flutter I48	527
Other chest pain R07.3	456
Atrial fibrillation and atrial flutter, unspecified I48.9	416
Left ventricular failure I50.1	163
Atherosclerotic heart disease I25.1	156
Acute subendocardial myocardial infarction I21.4	146
Unstable angina I20.0	97
Dressler syndrome I24.1	86
**Conditions potentially related to hospital care**	
Infection following a procedure, not elsewhere classified T81.4	592
Pneumonia, unspecified J18.9	313
Infection following a procedure, not elsewhere classified T81.4	281
Syncope and collapse R55	249
Other postprocedural disorders of circulatory system, not elsewhere classified I97.8	235
Pericardial effusion (noninflammatory) I31.3	207
Pulmonary embolism without mention of acute cor pulmonale I26.9	186
Dizziness and giddiness R42	145
Acute and subacute infective endocarditis I33.0	132
Urinary tract infection, site not specified N39.0	124
**Other conditions**	
Other specified surgical follow-up care Z48.8	703
Pleural effusion, not elsewhere classified J90	642
Care involving use of rehabilitation procedure, unspecified Z50.9	414
Other postprocedural respiratory disorders J95.8	387
Dyspnoea R06.0	202
Convalescence following surgery Z54.0	51
Malaise and fatigue R53	40
Other functional disturbances following cardiac surgery I97.1	35
Surgical follow-up care, unspecified Z48.9	34
Pain localized to upper abdomen R10.1	33
